# Critical Illness Associated Fatal Hypoglycemia in a Nondiabetic Male

**DOI:** 10.1155/2019/4790320

**Published:** 2019-05-22

**Authors:** Amit Toor, Anika Toor, Mahesh Krishnamurthy

**Affiliations:** Department of Internal Medicine, Easton Hospital, Easton, Pennsylvania, USA

## Abstract

We present here the case of a 55-year-old male who presented to the emergency department with severe abdominal pain and respiratory distress secondary to mesenteric ischemia. His critical illness on preexisting chronic kidney disease, previously undiagnosed alcoholic cirrhosis, and congestive heart failure led to a rare yet fatal consequence of refractory hypoglycemia. Critical illness associated hypoglycemia generally occurs as a result of high metabolic consumption with relative insulin excess and insufficient nutritional intake that is seen frequently in critically ill patients. This, along with reduced hepatic and renal gluconeogenesis seen in preexisting liver and renal disease, can cumulate to life-threatening hypoglycemia and is seen as a poor prognostic factor in the ICU setting.

## 1. Introduction

Carbohydrate metabolism and formation of glucose depends on a myriad of factors. The basic principle is dependent on nutritional intake, metabolic demand with relative insulin excess, and hepatic (to some degree also, renal) gluconeogenesis/glycogenolysis. Hypoglycemia in these nondiabetic patients can be diagnostically challenging and may indicate a poor prognosis especially in the ICU setting given severe nature of illnesses and insufficient nutritional intake. Various etiologies include medications and medication overdose, insulin producing tumors, history of gastric bypass surgery, alcohol use, critical illness, liver/renal disease, and adrenal insufficiency [1]. We present a case of a 55-year-old male with persistent, refractory hypoglycemia secondary to critical illness in the absence of Diabetes Mellitus (DM).

## 2. Case

A 55-year-old male with past medical history of congestive heart failure with ejection fraction of 30%, chronic kidney disease, atrial fibrillation, and alcohol abuse, presented with sudden onset of severe abdominal pain. Admission vitals were stable with the significant except oxygen saturation of 70% on room air. Labs on admission were significant for lactic acid of 5.3 mmol/L (Table 1), Acute Kidney Injury (AKI), and subtherapeutic INR of 1.5 on Coumadin. Urine toxicology screen was positive for cocaine use. CT scan of the abdomen was initially unremarkable. The patient was subsequently admitted to the ICU for severe acute hypoxic respiratory failure with potential for decompensation and clinical suspicion of mesenteric ischemia given his subtherapeutic INR in the setting of atrial fibrillation along with sudden onset of abdominal pain and elevated lactic acid without a clear cause. Cocaine abuse may have also been a contributing factor to sudden ischemia. A later repeat CT scan revealed nonspecific bowel wall thickening, and a transesophageal echocardiogram performed revealed an active left atrial thrombus, making mesenteric ischemia a higher differential. Due to the added possibility of sepsis and worsening renal function, there was no clear opportunity for surgical intervention, and conservative management was initiated through heparin drip and BiPAP showing initial clinical improvement. By hospital day 3, however, he suddenly became unresponsive. At that time, his labs revealed blood sugar of less than 10 mg/dl and worsening renal failure. The patient was aggressively managed and given multiple ampules of Dextrose 50% and infusion of Dextrose 5% was started (Table 2). Because of further decompensation of renal function, he was started on Continuous Renal Replacement Therapy (CRRT) as well. Despite these measures, his blood sugar continued to have multiple episodes reaching 40 mg/dl. His IV fluids were switched to Dextrose 10% drip and eventually to Dextrose 20% drip because of persistent episodes of hypoglycemia, also requiring intermittent IV Glucagon. The patient had no family or personal history of DM and HbA1c tested during hospitalization was 5.6%. Further testing including cortisol, pro-insulin, insulin, and C-peptide levels was within normal limits. A sulfonylurea screen was also negative. Liver function tests were within normal limits with exception of development of supratherapeutic INR of 4.4 despite not being on Coumadin, suggestive of underlying liver disease. Endoscopic Ultrasound did not reveal any pancreatic mass. The patient was subsequently transferred to a tertiary care center for further investigative work-up and management of persistent hypoglycemia. During this time, the patient had developed abdominal ascites. Paracentesis and analysis of the ascitic fluid was suggestive of underlying hepatic cirrhosis. Given the patient's history of severe alcohol abuse, it was more likely that the underlying hepatic illness was alcoholic liver cirrhosis. To confirm this, a biopsy was offered but the patient refused. It was determined that the patient's persistent hypoglycemia was likely secondary to critical illness along with and underlying background of severe hepatic and renal failure. The patient's overall prognosis was poor and his condition declined rapidly despite adequate advanced care and paracentesis. He refused any further intervention and opted for hospice care. An autopsy was not performed.

## 3. Discussion

The developing hypoglycemia secondary to underlying critical illness is seen in less than 1.5% of patients [2]. Despite this, there is a combination of factors that are commonly seen in the ICU setting that can contribute to its occurrence. The rise of the metabolic response in critically ill patients is seen as part of a defense mechanism in an attempt to maintain the nutritional demand of inflamed and vital organ systems [3, 4]. These mechanisms include increased sympathetic output, release of counter-regulatory pituitary and adrenal hormones, and increased rate of gluconeogenesis and glycogenolysis [5, 6]. With the development of fulminant hepatic disease and liver cirrhosis, the production of endogenous glucose is rapidly reduced. Without resolution or prevention of decompensation of hepatic disease, patients may develop hypoglycemia that is an indication of increased mortality risk. One study involving 4946 ICU patients who had developed hypoglycemia during their hospitalization revealed an in-hospital mortality of 36.6%, compared to 19.7% in nonhypoglycemic patients [7]. Acute renal failure can contribute to hypoglycemia as well. In the setting of uremia, there is reduced gluconeogenesis directly from the kidney as well as reduced production from the liver [8–10]. Also, with renal failure there is an increase in circulating insulin secondary to decreased clearance. This is generally seen when the glomerular filtration rate (GFR) is below 15–20 mL/min/1.73 m^2^ [11]. In patient's receiving renal replacement therapy, the sensitivity to insulin may improve, which can contribute to hypoglycemia as well [12]. In cases of severe cardiac failure in critically ill patients, it is theorized that consequent hepatic congestion may contribute to lower glucose output from the liver as well as reduced intestinal absorption [13, 14]. Alcohol abuse such as that seen in our patient also plays a major role in the development of hypoglycemia. The underlying mechanisms include alcoholic cirrhosis, malnutrition, and direct inhibition of gluconeogenesis [1]. In critically ill patients particularly in those with sepsis, adrenal insufficiency may be an important contributing factor as well. This can be screened with AM serum cortisol levels and further tested with Cosyntropin and ACTH testing [15]. Prior to attributing hypoglycemia to hepatic and renal failure in combination with severe illness, full investigative work-up should be done to exclude primary causes of hypoglycemia. This includes a complete review of patient medications and potential access to medications such as oral hypoglycemics. This can be confirmed with an oral sulfonylurea screen [1]. If insulinoma or any other form of endogenous hyperinsulinism is suspected, testing for this should be done in a fasting state if patient's status permits [1]. These tests include serum insulin, Proinsulin, and C-peptide [16]. Management of secondary hypoglycemia should be emphasized on appropriate nutritional repletion and attempted at resolution of acute illness as well as compensation for decompensated chronic diseases. There are no reliable simple equations for determination of energy requirements in such patients, given that their energy expenditures are highly variable. One of the only reliable determinants of energy expenditure and requirement is through indirect calorimetry [17]. Determination of this can guide nutritional repletion in enteral or parenteral forms. If a primary cause is identified, medical or surgical options should be explored for that specific etiology.

## 4. Conclusion

Patients with critical illness especially those with underlying advanced liver/renal disease, malnutrition, and advanced age may develop profound hypoglycemia even in the absence of diabetes. Recognition of malnutritional states and insufficient nutritional intake in the background of decompensated liver and kidney disease is important to prevent such life-threatening hypoglycemia. Such patients require frequent monitoring of blood sugar levels and adequate enteral or parenteral nutritional support to accommodate the increased metabolic demand and reduced gluconeogenesis. Close monitoring and prompt support for decompensated chronic illness may benefit in terms of overall survival.

## Figures and Tables

**Table 1:** Outline of pertinent lactic acid, liver enzymes, and INR throughout hospitalization.

	Day 1	Day 2	Day 3	Day 4	*Day 8*	Day 9	Day 10
Lactic Acid (mmol/L)	5.3	3.6	6.7	1.8	1.8	3.0	3.3

AST/ALT (U/L)	30/71	31/70	91/243	122/232	82/99	85/120	81/97

INR	1.5	1.5	2.5	2.1	3.4	6.9	4.4

**Table 2:** Outline of pertinent lowest glucose levels throughout hospitalization along with correction measures taken.

	Day 1	Day 2	Day 3	Day 4	*Day 10*	Day 11	Day 12	Day 13
Glucose (mg/dl)	88	78	<10	98	<30	30	46	36

Glucose/Glucagon therapy	-	-	Multiple ampules of D50 D5NS IV	D5NS IV	Multiple ampules of D50 D5NS to D10NS IV Glucagon IV	D10NS IV	D10NS IV Glucagon IV	D20NS IV Glucagon IV

D50: Dextrose 50% ampule, D5NS: Dextrose 5% in normal saline, D10NS: Dextrose 10% in normal saline, D20NS: Dextrose 20% in normal saline.
